# Biomechanical comparison of five cannulated screw fixation strategies for young vertical femoral neck fractures

**DOI:** 10.1002/jor.24881

**Published:** 2020-10-23

**Authors:** Dajun Jiang, Shi Zhan, Lei Wang, Lewis L. Shi, Ming Ling, Hai Hu, Weitao Jia

**Affiliations:** ^1^ Department of Orthopedic Surgery Shanghai Jiao Tong University Affiliated Sixth People's Hospital Shanghai People's Republic of China; ^2^ Orthopedic Biomechanical Laboratory, Department of Orthopedic Surgery Shanghai Jiao Tong University Affiliated Sixth People's Hospital Shanghai People's Republic of China; ^3^ Department of Orthopaedics University of Chicago Medical Center Chicago Illinois USA

**Keywords:** cannulated screw fixation, interfragmentary motion, patient‐specific finite element analysis, vertical femoral neck fractures

## Abstract

Vertical femoral neck fractures in patients younger than 65 years of age often require hip‐conserving surgeries. However, traditional fixation strategies using three parallel cannulated screws often fail in such patients due to an unfavorable biomechanical environment. This study compared different cannulated screw fixation techniques in patients via patient‐specific finite element analysis with linear tetrahedral (C3D4) elements. Forty vertical femoral neck fracture models were created based on computed tomography images obtained from eight healthy participants. Five different fixation strategies: alpha, buttress, rhomboid, inverted triangle, and triangle were assessed in walking status. Biomechanical parameters including stiffness, interfragmentary motion in two directions (detachment and shearing), compression force, and maximal implant stress were evaluated. The mean relative coefficient of strain distribution between the finite element analysis and experiment was from 0.78 to 0.94. Stiffness was highest (*p* < .05) in the buttress group (923.1 N/mm), while interfragmentary motion was lowest (*p* < .05) in the alpha group. Maximal stress was highest (*p* < .05) in the buttress group and lowest in the alpha group. Shearing values were significantly lower in the alpha group than in the rhomboid group (*p* = .004). Moreover, Shearing values were significantly higher (*p* = .027), while detachment values were significantly lower (*p* = .027), in the inverted triangle than in the triangle group. Clinical significance: Our results suggest that alpha fixation is the most reliable and biomechanically efficient strategy for young patients with vertical femoral neck fractures. Regular and inverted triangular fixation strategies may be suitable for fractures of different skeletal constructions due to antidetachment/shearing abilities.

## 1 INTRODUCTION

Vertical femoral neck fractures in patients younger than 65 years of age often require hip‐conserving surgeries^1^; however, such procedures remain challenging for orthopedic surgeons due to their high‐energy nature,^2^ general vulnerabilities in the vasculature,^3^ and an unfavorable biomechanical environment. Anatomic reduction, thorough stable fixation, and primary healing are necessary for reducing the risk of avascular necrosis and non‐union in these patients. However, the optimal fixation strategy for this fracture type remains controversial.^4^

According to a web‐based survey conducted in 2014,^4^ sliding hip screws and cannulated screws are the two most commonly utilized fixation devices for vertical femoral neck fractures. Recently, sliding hip screws have been criticized^5^, ^6^ due to their association with an increased risk of osteonecrosis. Thus, cannulated screws remain the most promising and commonly used devices^1^ because of their minimal invasiveness, easy handling, and ability to induce dynamic compression. Unfortunately, due to the adverse nature of vertical femoral neck fractures, traditional methods utilizing three cannulated parallel screws are associated with high rates of mechanical failure (19%) and osteonecrosis (14%) in patients with Pauwels type III vertical femoral neck fractures.^7^ Given that these methods are widely accepted amongst surgeons, it remains necessary to identify more efficient screw fixation strategies that can reduce the rates of such complications.

Recently, researchers have successfully modified fixation techniques by adding a fourth screw or augmenting the fixation system with a buttress plate.^8^, ^9^ However, few studies have systematically compared biomechanical outcomes among the available fixation methods. To provide guidance for clinical practice, the present study aimed to compare different cannulated screw fixation techniques in patients with vertical femoral neck fractures and to illustrate the detailed biomechanical properties of these techniques via patient‐specific finite element analysis (FEA).

## 2 MATERIAL AND METHODS

### 2.1 Models establishment

**Figure 1:**
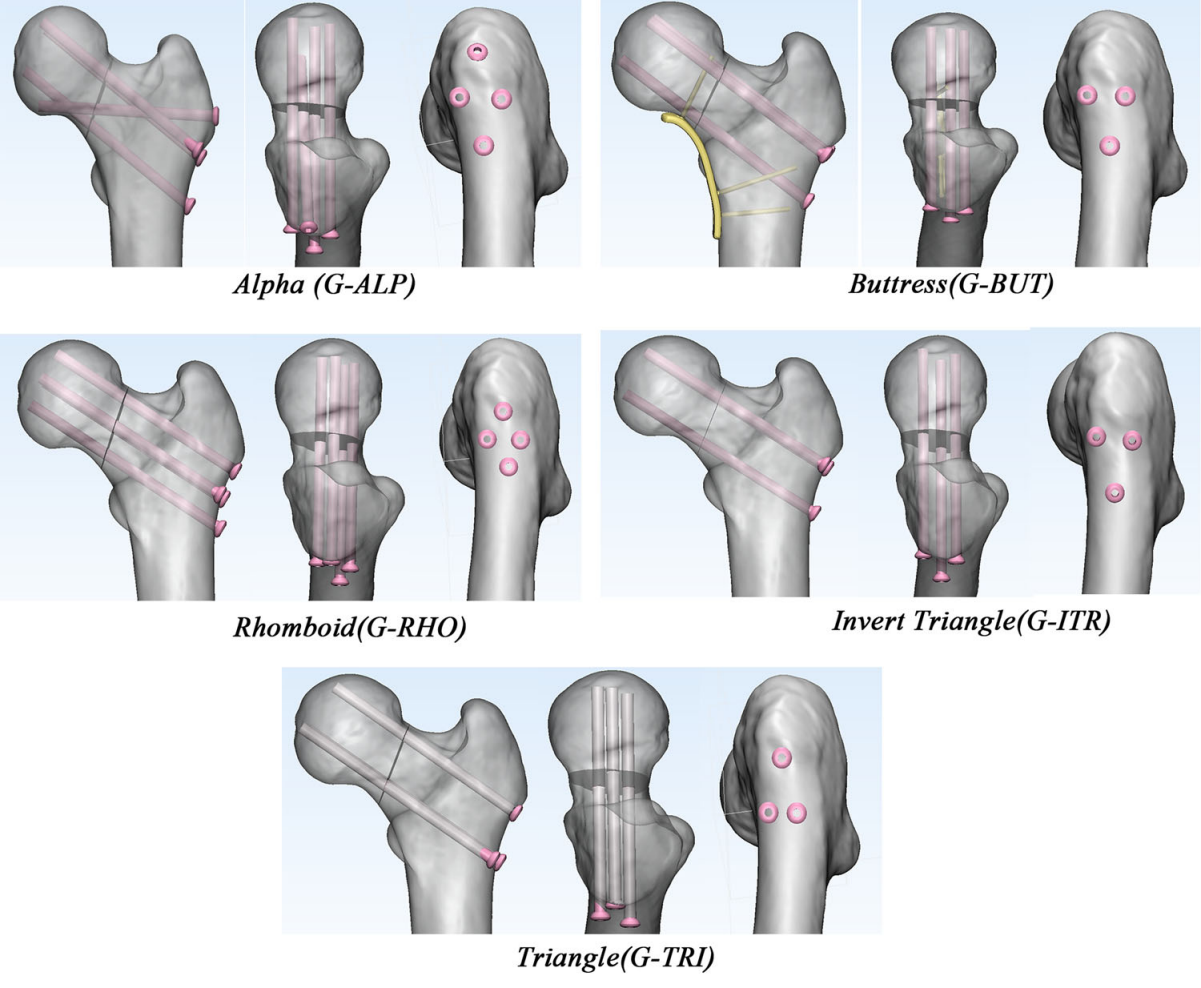
The five groups of fixation models in anterior view, superior view, and lateral view from left to right, including group alpha (G‐ALP), buttress (G‐BUT), rhomboid (G‐RHO), inverted triangle (G‐ITR), triangle (G‐TRI) [Color figure can be viewed at wileyonlinelibrary.com]

Eight healthy volunteers ranging in age from 20 to 55 years without any history of hip fracture, metabolic bone disease, or general comorbidities were recruited for the present study (Table 1). Mimics software (Version 19.0; Materialise) was used to develop patient‐specific three‐dimensional (3D) models with a modified Pauwels angle of 70° based on 0.625 mm thick computed tomography (CT) images. The models were then osteotomized using 3‐Matic software (Version 11.0; Materialise). We assessed the effects of five different internal fixation strategies in each model (Figure 1): (1) three inverted parallel screws plus one off‐axis screw, arranged in an “alpha” configuration (group ALP [G‐ALP]); (2) three inverted parallel screws plus one buttress plate strengthening the calcar (G‐BUT); (3) four parallel screws arranged in a “rhomboid” configuration (G‐RHO); (4) three parallel screws with an inverted triangular construction (G‐ITR); (5) three parallel screws with a triangular construction (G‐TRI).

**Table 1:** Baseline information

Patient	Age	Gender	Height (m)	Weight (kg)	BMI (kg/cm^2^)	HU^a^	Neck length (mm)^b^	Neck thickness (mm^2^)^c^	NSA
1	29	F	1.65	50	18.37	491.66	34.21	522.13	121.76
2	30	F	1.55	80	33.29	410.5	32.9	390.86	121.88
3	55	F	1.58	55	22.03	435.58	38.16	586.96	126.81
4	21	M	1.7	70	24.22	372.94	41.57	578.96	137.31
5	48	M	1.73	85	28.4	563.72	33.16	947.71	124.8
6	54	M	1.7	70	24.22	409.66	34.68	789.88	122.12
7	30	M	1.82	100	30.19	533.36	35.15	1003.69	124.12
8	35	M	1.78	75	23.67	581.38	35.45	928	137.55

Abbreviations: BMI, body mass index; NSA, femoral neck shaft angle.

^a^ Hu indicates average Hu value in the fitted ovoid region of the middle femoral neck. A Hu value above 262 can be used to confirm the absence of osteoporosis according to the previous article.

^b^ Neck Length was defined as the distance between femoral head center and femoral neck base along with femora neck axis.

^c^ The definition of neck thickness was the area of the narrowest section of femoral neck.

To control the essential confounding variable of screw position, all the cannulated screws were implanted according to the same standard criteria, which has been well‐studied and established in previous studies.^10^, ^11^, ^12^, ^13^, ^14^ For the parallel cannulated screws, the directions were along the femoral neck axis which was automatically calculated in MATLAB (The MathWorks).^15^ The parallel screws were positioned dispersedly,^10^, ^11^ at 2.5 mm to the cortex of the femoral neck,^12^, ^13^ and 5 mm distal to the subchondral bone in the femoral head.^14^ The off‐axis screw in G‐ALP was implanted at 5 mm proximal to the most prominent part of the great trochanter (to prevent soft tissue irritation due to screw protruding) and targeted at the inferior femoral head‐neck junction (to provide more favorable bone mass for screw purchase).

The constructs were all created in SolidWorks2017 (DS SolidWorks Corp.) using 6.5‐mm cannulated screws (Stryker) and a 6‐hole, 2.7‐mm AO locking plate with 2.7 mm diameter locking screws (Depuy‐Synthes). The locking screws were all fixed using unicortical fixation, and were 30 mm (1st hole), 40 mm (5th hole), and 30 mm (6th hole) in length. Thus, 40 models were generated across the eight participants.

### 2.2 FEA validation

**Figure 2:**
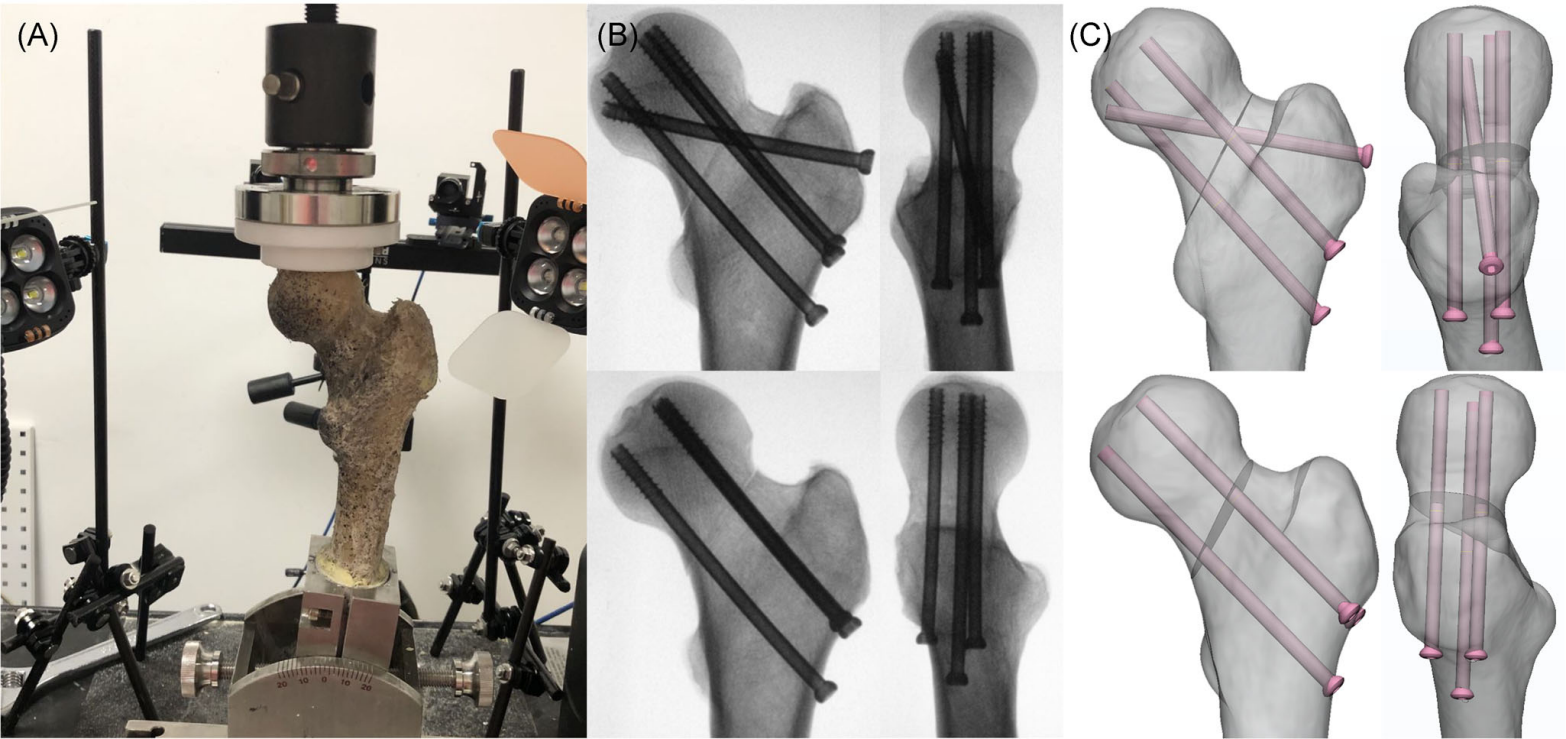
(A) Intact and fixation models were tested under 237.7% body weight with Instron recording the load‐displacement curve and VIC‐3D recording the strain distribution. (B and C) In the validating experiment, the models between finite element analysis and biomechanical experiment were consistent with the help of 3‐D printed guiding and osteotomy templates during surgical process [Color figure can be viewed at wileyonlinelibrary.com]

The donor was 160 cm in height, 50 kg in weight, and absent of any reported musculoskeletal disorders. The pair of cadaver bones harvested from the donor was used in the two‐step experiment of the validation test. All the bones were potted with polymethyl methacrylate 60 mm distal from the lesser trochanter and loaded on the head with 1188.5 N (approximately 237.7% body weight) at an angle of 7° relative to the femur shaft axis^16^ in the Instron test system (Instron) to simulate the mechanical status during walking^17^ (Figure 2A). The noncontact strain measurement system, VIC‐3D (XR‐9M; Correlated Solutions Company) was used to record strain distribution at the surface. This system is based on the principles of continuum mechanics^18^ and can capture consecutive images of the surface of a tested object during the deformation period. Finally, the displacement and strains of the speckles on the surface can be calculated precisely. First, the intact bones were tested mechanically to validate the material assignment method, boundary condition, meshing type, and quality. Then, the bones were osteotomized with a 70° Pauwels angle and fixed with screws in the G‐ITR and G‐ALP configurations separately. To ensure the consistency of the model between the experiment and the simulation, a 3D‐printed guiding plate was applied to ensure the identical position (Figure 2B,C) of the fracture line and cannulated screws. The surgical bones were then tested to validate the assignment of the contact interfaces. Principal strain and interfragmentary motion (IFM) were recorded via VIC‐3D, and used for comparison with FEA results.

The FEA models and boundary conditions were established in accordance with the mechanical experiment. Intact and surgical bones were all meshed to 1‐mm equal‐sized facets according to the results of previous mesh convergence tests on similar models^19^ and checked for quality in Hypermesh 13.0 (Altair Engineering). The models were then meshed with 4‐node linear (C3D4) and second‐order tetrahedron (C3D10) elements separately and were all exported into Abaqus 6.13 (Simulia Corp.) for further FEA. All bone and implant models were assumed to behave with linear elastic properties. The apparent density (*ρ*), Young's modulus (*E*), and Poisson's ratio of each element were assigned based on the Hu value in the CT scans according to the following formula,^20^ which made a distinction between cancellous and cortical bone:

$$\rho \;(g/{\mathrm{cm}}^{3})=0.000968\times \mathrm{HU}+0.5,$$

$$\text{If}\rho <\frac{1.2\;g}{{\text{cm}}^{3}},\;E=2014,\;\rho^{2.5}\;\left(\mathrm{MPa}\right),\;\nu =0.2,$$

$$\text{If}\rho <\frac{1.2\;g}{{\text{cm}}^{3}},\;E=1763,\;\rho^{3.2}\;\left(\mathrm{MPa}\right),\;\nu =0.32,$$

**Figure 3:**
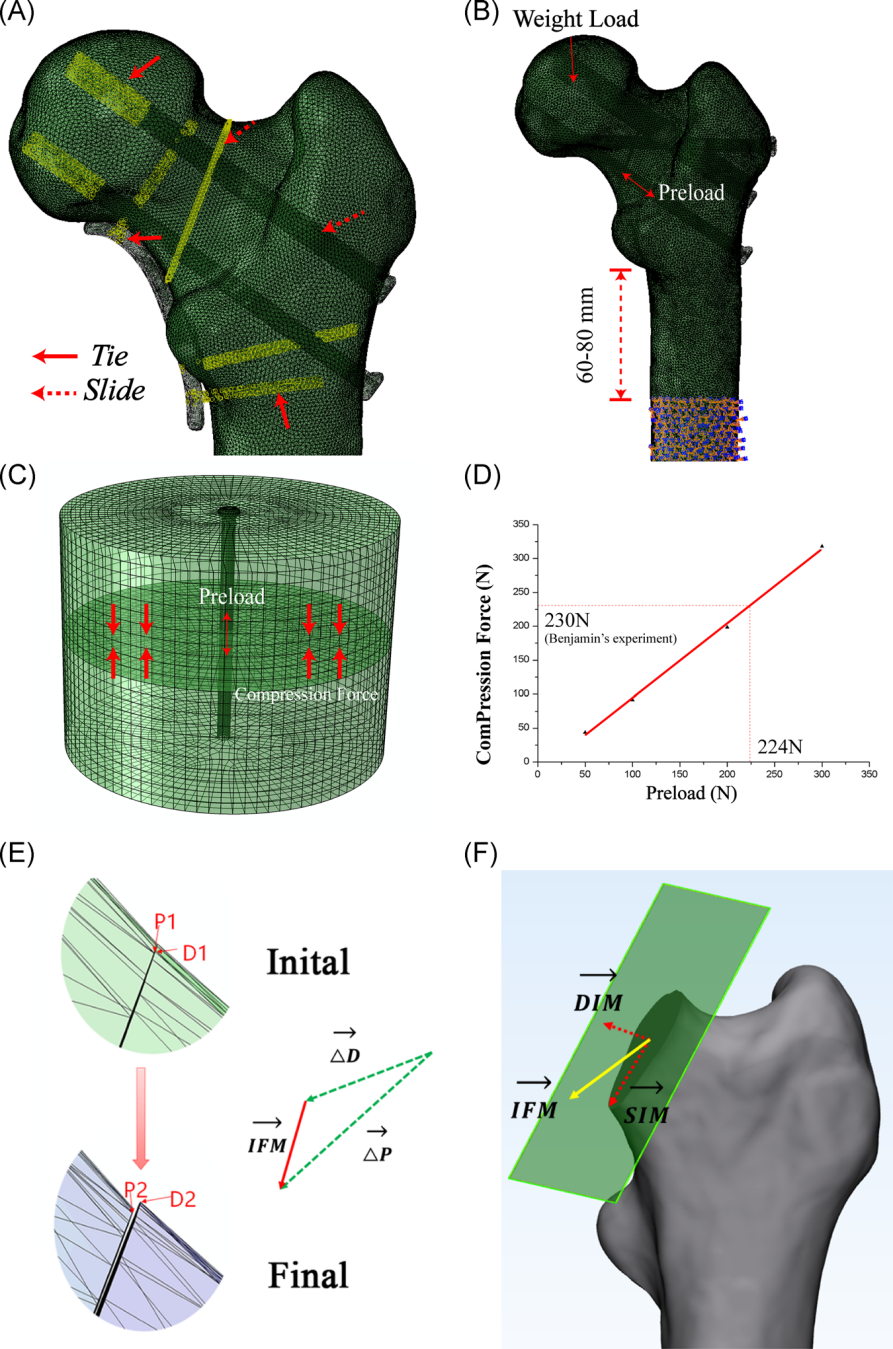
The schematic diagram of finite element analysis. (A) Tie contacts (solid arrow) were assigned to thread‐bone, locking screw‐plate, locking screw‐bone junctions, while slide contacts (dotted arrow) were assigned to shaft‐bone, and fracture surface. (B) A 224 N preload was applied first to the cannulated screw, then 237.7% body weight load was applied to the femoral head. (C and D) The finite element analysis model was created in accordance with the boundary conditions in Benjamin's experiment.^24^ A series of 50, 100, 200, and 300 N bolt loads were exerted on the 6.5‐mm Stryker cannulated screw to find an appropriate value. According to the regression analysis, it was found that a preload of 224 N can generate the same compression force (230 N) in the previous study. (E and F) The schematic diagram of interfragmentary motion (IFM) algorithm. (E) All nodes in the proximal and distal fracture surface were selected for analyzing. The node of the distal fracture surface closest to a selected proximal node was assumed as paired nodes. For each matched node (e.g., P1 and D1) moved to the final position (P2 and D2), the vector of IFM was defined as the displacement of the proximal node relative to the distal node, and the absolute value was used for comparison $\mathrm{IFM}=\left|\vec{\Delta_{D}}-\vec{\Delta_{P}}\right|$. (F) IFM was further divided into two components either in the shear direction (shear interfragmentary motion [SIM]) or in the detached direction (detached interfragmentary motion [DIM]) [Color figure can be viewed at wileyonlinelibrary.com]

All cannulated screws were assigned as titanium (Ti‐6L‐4V), with Young's modulus (*E*) of 110,000 MPa and Poisson's ratio of 0.3.^21^, ^22^ Thread–bone interfaces were tied while shaft‐bone and fracture interfaces were assigned as slide contact with a frictional coefficient^23^ of 0.46 and 0.3, respectively (Figure 3A). To simulate the dynamic compression force of the cannulated screws, an extra 224 N preload (Figure 3B) was applied to the middle of the screw shaft using the bolt load in Abaqus software. This was estimated as there are few studies describing the exact value of the preload. Consequently, in our preliminary experiment (Figure 3C), we applied a series of 50, 100, 200, and 300 N bolt loads on cannulated screws during FEA to achieve identical compression forces to that of 6.5 mm Stryker screws from a previous study^24^ using a linear regression method (Figure 3D).

Strain distribution was compared between FEA and VIC‐3D in intact and osteosynthesis bones. The regions captured by VIC‐3D were automatically matched with the identical regions in FEA using the iterative closest point (ICP) algorithm^25^ in MATLAB software. The distribution of maximal as well as minimal principal strain, stiffness, and IFM were compared between the simulation and the experiment.

**Figure 4:**
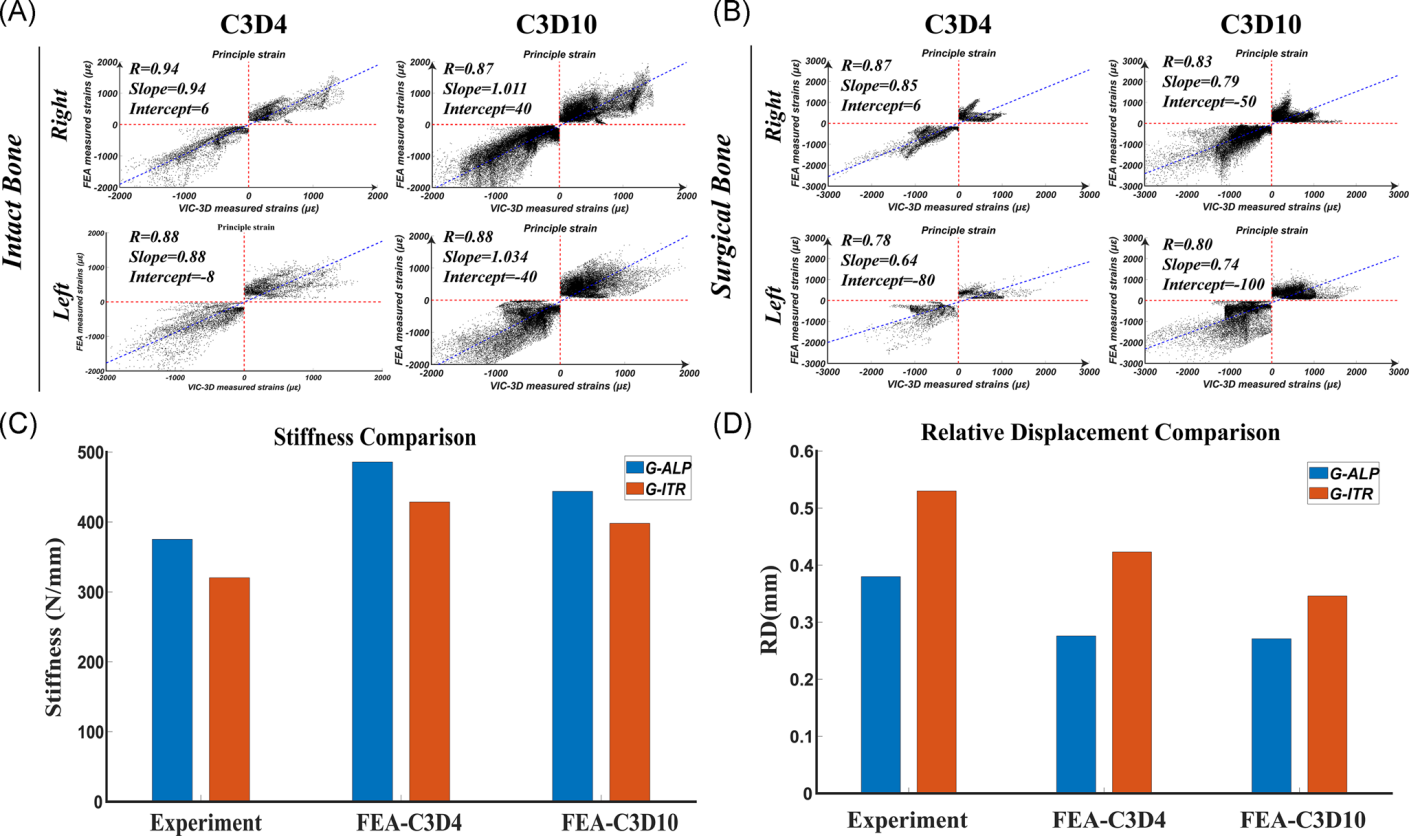
Results of the validation experiment. (A and B) Lagrange principal strain of each paired node in finite element analysis (FEA) simulation were all linear correlated with that in VIC‐3D test. (A)The relative coefficients in right and left legs of the same donor were 0.94, 0.88 for C3D4 models, and 0.87, 0.88 for C3D10 models. (B) The relative coefficients in surgical bones were 0.87, 0.78 for C3D4 models, and 0.83, 0.80 for C3D10 models. (C and D) In comparison between G‐ALP and G‐ITR, the tendencies of stiffness and interfragmentary motion were similar between experiment and FEA (both of C3D4 and C3D10) [Color figure can be viewed at wileyonlinelibrary.com]

In the preliminary validation test, C3D4 and C3D10 FEA models had a similar mean relative coefficient (C3D4: 0.78–0.94 vs. C3D10: 0.80–0.88) (Figure 4A,B) and the same IFM as well as stiffness tendencies (Figure 4C,D) with the biomechanical experiments. Consequently, FEA with C3D4 elements were sufficient for mechanical comparisons in the present study and were used in the further patient‐specific FEA study.

### 2.3 Patient‐specific FEA simulation

Following the validation experiment, patient‐specific FEA was performed in all the 40 models with C3D4 meshes using the abovementioned procedure. There were approximately 500,000 elements (from 430,826 to 542,864) and 100,000 nodes (from 93,492 to 117,661) in each model. Additionally, tie contacts were assigned to the plate‐screw and screw‐bone interfaces in the G‐BUT models. All the models were subjected to 237.7% body weight in line with the femoral mechanical axis. Parameters including stiffness, IFM, compression force, and implant stress were comprehensively analyzed. Stiffness was calculated by dividing patient‐specific load by the displacement of the applying node. The IFM of each node was calculated using a previously described formula (Figure 3E)^26^, ^27^ and was further decomposed into two components either in the shear direction (shearing interfragmentary motion [SIM]) or detached direction (detachment interfragmentary motion [DIM]) (Figure 3F). Compression force was calculated based on the mean stress in the direction of the normal vector after preloading multiplied by the surface area of the fracture. Data extracted from the FEA were primarily tested for normality using the Kolmogorov–Smirnov test. Randomized block one‐way analysis of variance test was used for comparison among five groups and paired *t* test was used for comparison between just two groups.

## 3 RESULTS

In the validation study, we observed a significant linear correlation in the strain distributions between the FEA and VIC‐3D results (*p* = .000). The mean relative coefficient was 0.94, 0.88 for intact bones and 0.87, 0.78 for surgical bone. The same IFM as well as stiffness tendencies in two fixation groups were observed between the experiment and FEA (Figure 4C,D).

**Figure 5:**
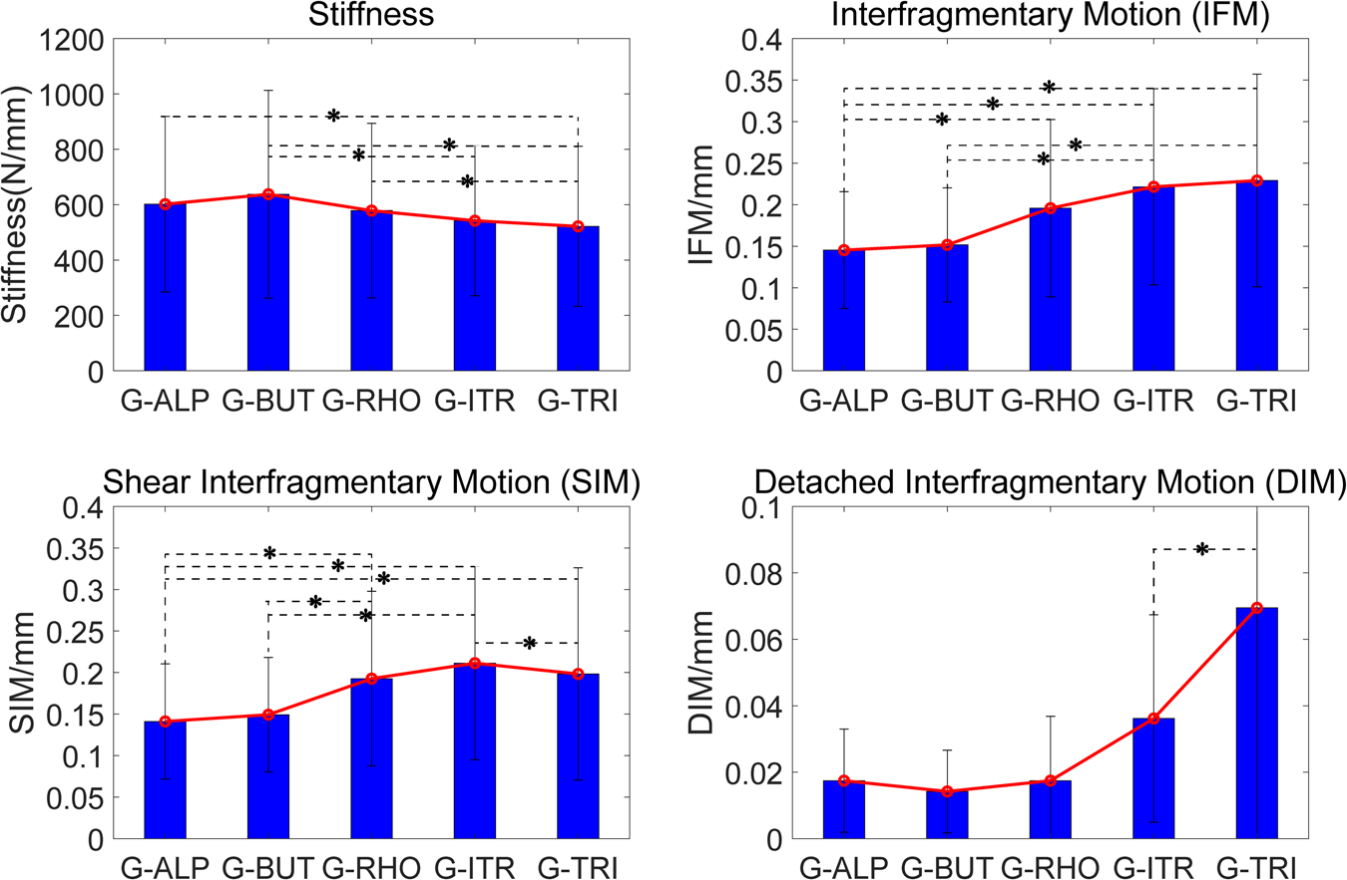
Comparison of stiffness, interfragmentary motion (IFM), shear interfragmentary motion (SIM), and detached interfragmentary motion (DIM) among five groups. G‐BUT were the highest stiffness (923.1 N/mm) devices, while G‐ALP and G‐BUT have similarly lowest IFM, SIM, DIM among these groups [Color figure can be viewed at wileyonlinelibrary.com]

Among the five internal fixation groups, stiffness was highest in G‐BUT (923.1 N/mm), and the lowest IFM value was observed in G‐ALP (Figure 5). Furthermore, IFM values in G‐ALP (0.072 ± 0.031 mm) were significantly (*p* < .05) lower than those in the G‐RHO, G‐ITR, and G‐TRI groups, while those in G‐BUT (0.080 ± 0.028 mm) were significantly (*p* < .05) lower than those in G‐ITR and G‐TRI. G‐ALP and G‐BUT were the two most stable techniques in terms of SIM.

**Figure 6:**
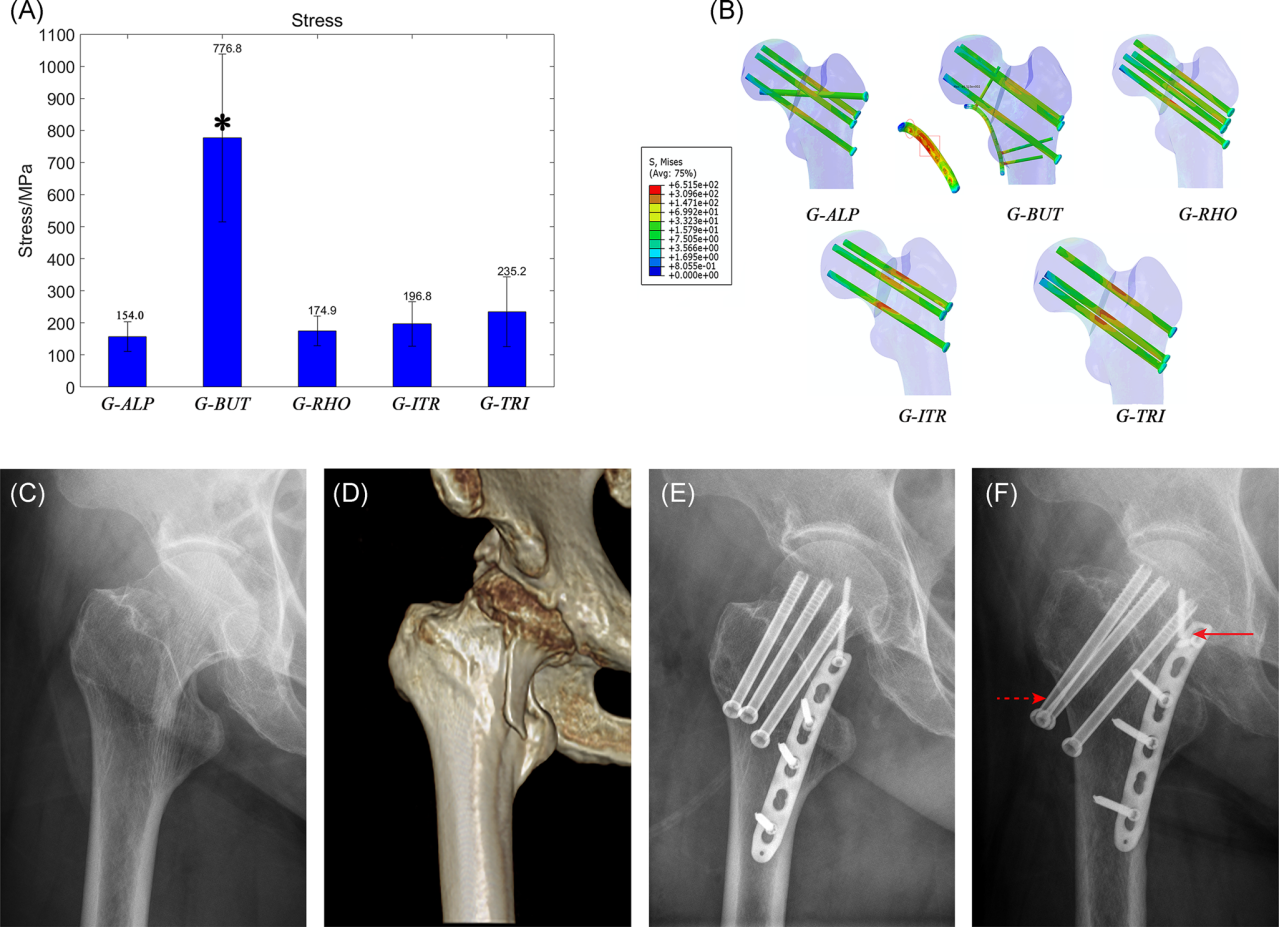
Maximal stress of internal fixation devices. (A) A comparison of maximal stress value of five groups. This value was significantly (*p* < .05) highest (776.8 + 244.6 MPa) in G‐BUT but lowest (157.4 + 40.5 MPa) in G‐ALP. (B) Von Mises Stress distribution in devices of five groups according to the same gradience. G‐ALP fixation has the smallest high‐stress regions (red color). There are two special stress concentration regions in buttress plate which indicate mechanical pitfall for such strategy. (C and D) Preoperative radiograph of a 64‐year‐old woman showing vertical femoral neck fracture. (E) Radiograph on the second day after this patient was treated with G‐BUT. (fF) Postoperative radiograph approximately 15 months after fixation showed a breakage at the plate‐screw junction (solid arrow), screw withdraw (dotted arrow), and femoral neck shortage. The breakage site was the same as the stress concentration region in our simulation [Color figure can be viewed at wileyonlinelibrary.com]

Maximal stress was significantly (*p* = .000) higher in G‐BUT (776.8 ± 244.6 MPa) than in each of the remaining four groups, while it was lowest in G‐ALP (154.0 ± 40.5 MPa) (Figure 6A). In addition, several sites of stress concentration were detected in G‐BUT, including at the curvature and locking plate‐screw junction (Figure 6B).

**Figure 7:**
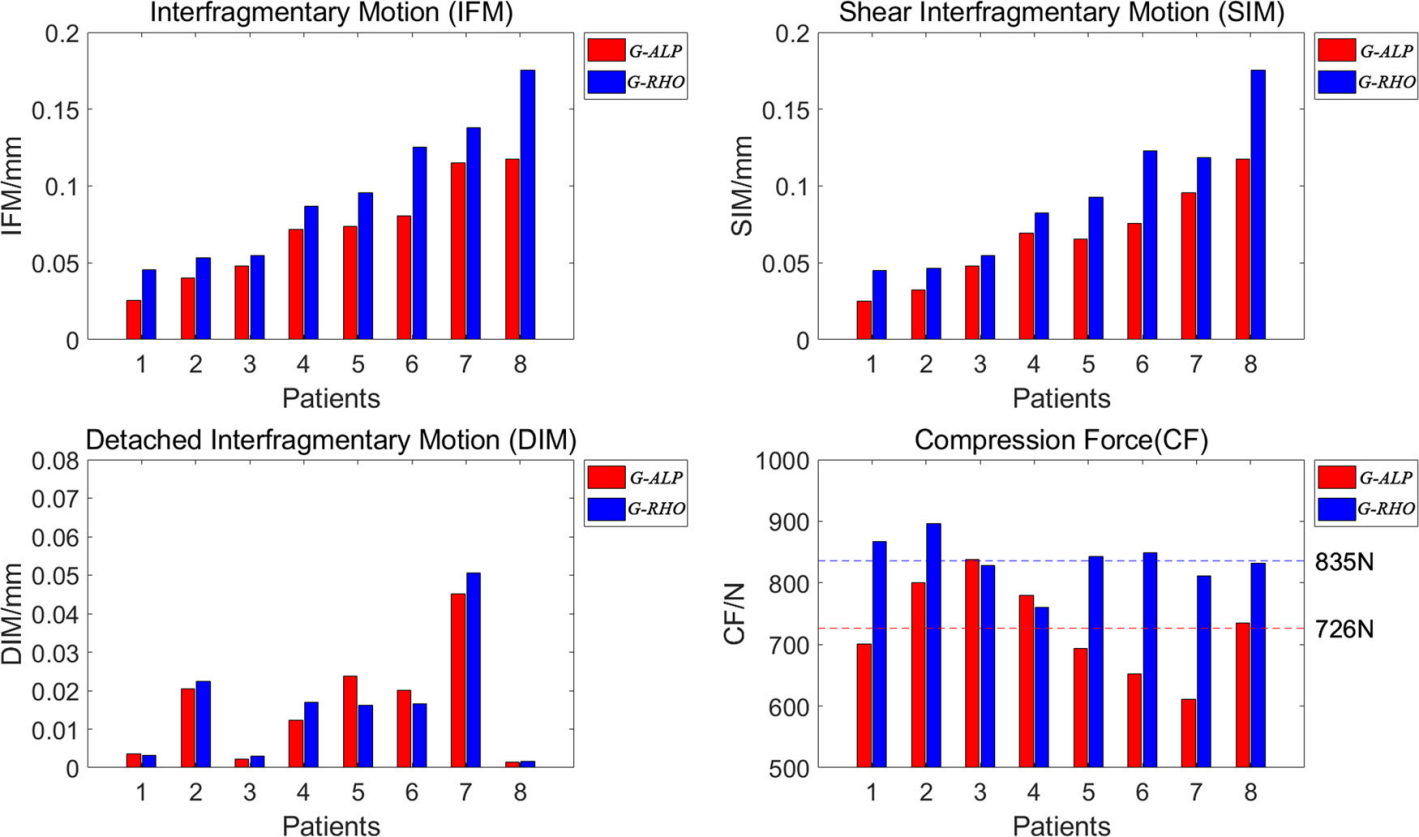
A detailed comparison of interfragmentary motion (IFM), shear interfragmentary motion (SIM), detached interfragmentary motion (DIM), and compression force (CF) between G‐RHO and G‐ALP). G‐ALP had significantly lower compression force (*p* = .008), similar DIM values (*p* = .988), and significantly lower IFM (*p* = .004) and SIM values (*p* = .004) than G‐RHO [Color figure can be viewed at wileyonlinelibrary.com]

We then compared the mechanical behavior of the off‐axis screw between G‐ALP and G‐RHO (Figure 7). Our analysis indicated that compression force was significantly lower in G‐ALP than in G‐RHO (*p* = .008), while DIM values were similar (*p* = .988). However, IFM (*p* = .004) and SIM values (*p* = .004) were also significantly lower in G‐ALP than in G‐RHO.

**Figure 8:**
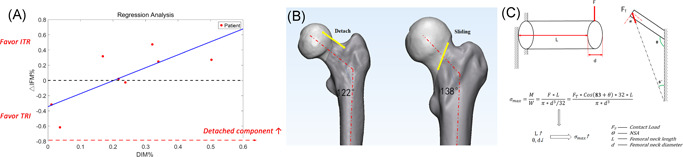
Mechanical differences of regular and invert triangle screws fixation strategies. (A) ΔIFM% ((IFM(G‐TRI) − IFM(G‐ITR))/IFM(G‐ITR))) has a significant (*p* < .05) linear correlation with DIM% (DIM/IFM), which means that as detached proportion of IFM became larger, the biomechanical benefits of G‐ITR became increasingly prominent. (B) Analysis of the bone structures of the patients suffering from the greatest detached and shear force. The femur on the left has a typically thin femoral neck (390.66 mm^2^) with the lowest neck‐shaft angle (137.55°) which was better to be fixed by ITR whereas the one on the right has a thick neck (928.00 mm^2^) with the highest neck‐shaft angle (137.55°) which was better to be fixed by G‐TRI. (C) The derived formula used to demonstrate the phenomenon that the detached force increased as the thickness of femoral neck and the neck‐shaft angle decreasing. M, moment; W, section factor. Note that the angle between anatomic and mechanical femoral axis was assumed as a mean value of 7°. IFM, interfragmentary motion [Color figure can be viewed at wileyonlinelibrary.com]

We also performed detailed comparisons between G‐ITR and G‐TRI. Overall, stiffness (*p* = .143) and IFM values (*p* = .766) were similar between G‐ITR and G‐TRI. However, G‐TRI exhibited significantly lower (*p* = .027) SIM values, while G‐ITR exhibited significantly higher (*p* = .027) DIM values. The percentage of IFM difference (ΔIFM%) also exhibited a significant (*P* = .027) linear correlation with the percentage of DIM (DIM%) (Figure 8A).

## 4 DISCUSSION

In the preliminary validation study, the correlation coefficient (*R* = 0.78‐0.94) for the results of the FEA and VIC‐3D analyses was satisfactory and within the range of values when compared to previous studies (*R* = 0.74–0.96).^28^, ^29^ The models with C3D4 performed no worse than those with C3D10 in terms of principal strain distribution, showing similar correlation coefficient (*R*‐value). Quadratic tetrahedral elements (C3D10) modeling can obtain more accurate values owing to denser nodes compared to linear tetrahedral (C3D4), which was confirmed in our test with the value of the slope closer to one. The results of differences between G‐ALP and G‐ITR in stiffness and IFM were the same for the two element types which was consistent with the mechanical experiment. Besides, the C3D4 model has been commonly used in many studies,^9^, ^30^, ^31^, ^32^ and it was proved to be similar to C3D10 models in accuracy under axial deformation (error: 2.7% vs. 2.8%).^33^ Consequently, FEA with C3D4 elements were sufficient for mechanical comparisons among different devices under axial loading in the present study.

The present study investigated the biomechanical properties of different cannulated screw fixation strategies for relatively young patients with vertical femoral neck fractures. Our analysis did not reveal a positive relationship between stiffness and IFM. Note that, stiffness is an engineering term that may not accurately reflect stability around the fracture site, whereas reduced IFM may reflect true “stability”^34^ The latter affects hard callus bridging across the fracture site according to Perren's strain theory, which is directly related to primary bone healing. Unfortunately, previous biomechanical studies have rarely mentioned this parameter,^17^ which may be difficult to measure accurately with current technologies.

Although our findings indicate that the greatest stability (i.e., least IFM) was achieved in G‐ALP and G‐BUT, significant stress concentration was detected in G‐BUT. Given that no such findings were observed in G‐ALP, this technique may be associated with more desirable biomechanical properties than G‐BUT. In accordance with the results of in vitro biomechanical analyses, augmentation with a buttress plate was associated with the highest stiffness values in our study.^8^ Most importantly, similarly lowest IFM was observed in the G‐BUT, which may explain the observed improvements in the clinical union rate (89%) when compared with those for traditional methods using three cannulated screws.^35^ Although buttress plates exhibit an exceptional anti‐shearing ability, few researchers have commented on its outstanding ability to withstand detachment even under an ordinary compression force. The detachment force in the superior part of the fracture surface may be transferred to the plate‐screw junction via the proximal locking screw. This force can also be ascertained based on the residual stress concentration at the plate‐screw junction (Figure 6B). Unfortunately, the stress concentration of the plate‐screw junction represents a major mechanical pitfall of this technique, given that it is associated with an increased risk of breakage and subsequent fixation failure^35^ (Figure 6C–F). Furthermore, applying a buttress plate may exert detrimental effects on blood supply to the femoral neck due to additional dissection. This may endanger the inferior retinacular artery, which plays an important role in perfusing the femoral head following femoral neck fractures.^36^, ^37^ Moreover, application of a buttress plate in patients with femoral neck fractures (especially those with the subcapital type) increases the risk of impingement^35^, ^38^ as the hip flexes, potentially leading to significant complications such as osteoarthritis. Given these complications, augmentation using a buttress plate may not be ideal in patients with femoral neck fractures. Nonetheless, buttress plates may be helpful in patients with osteoporotic or comminution when adequate bone purchase and compression force cannot be achieved using cannulated screws.

**Figure 9:**
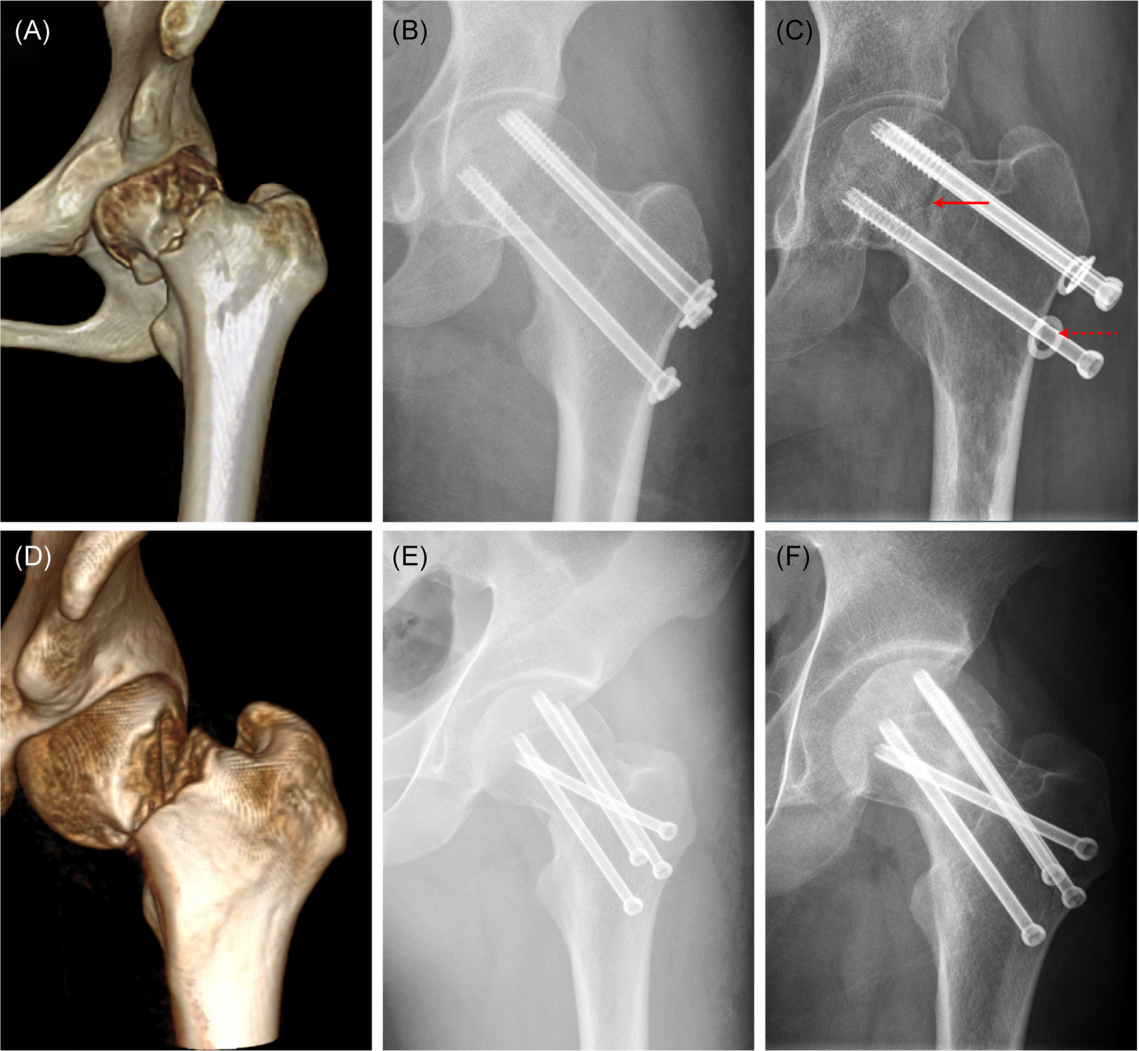
Clinical observation of alpha and invert triangle fixation strategies. (A) Preoperative computed topography of a 34‐year‐old female with a Pauwels type‐3 femoral neck fracture. (B) Radiograph on the second day after this patient was treated with traditional three cannulated screw. (C) Postoperative radiograph approximately 14 months after fixation showing non‐union (solid arrow) and screw withdraw (dotted arrow). (D) Preoperative computed topography of another 26‐year‐old male with a vertical femoral neck fracture. (E) Radiograph on the second day after this patient was treated with cannulated screws with an alpha configuration. (F) Radiograph showed fracture union at 13 months postoperatively [Color figure can be viewed at wileyonlinelibrary.com]

To further investigate the biomechanical properties at the off‐axis screw, we compared IFM between G‐RHO and G‐ALP, decomposing IFM into shearing (SIM) and detachment (DIM) components. In accordance with previous hypotheses,^39^, ^40^ we observed compromised compression force in G‐ALP due to the lack of parallelism, although these decreases in compression force did not compromise DIM in our study. In contrast, anti‐shearing ability was significantly greater in G‐ALP than in G‐RHO, indicating that the biomechanical effect of the off‐axis screw is to improve anti‐shearing stability. This unique biomechanical advantage can be explained as follows: First, the off‐axis screw is more likely to be perpendicular or angulate upward to the fracture plane, which can neutralize the sliding effect^6^ caused by three angulated parallel screws. Thus, the technique may confront shearing forces more efficiently. Second, bone quality is much better around the calcar than around Ward's triangle of the femoral head, leading to better bone purchase for techniques utilizing an off‐axis screw. Third, the off‐axis screw acts as a lever to transfer the bending moment from the femoral head to the calcar, thereby enhancing cortical support.^41^ Furthermore, in contrast to the RHO technique, the off‐axis screw used in the ALP technique will not increase stress at the lateral wall, which can reduce the risk of iatrogenic fractures. In accordance with previous findings, the biomechanical properties observed in the present study indicate that techniques utilizing an off‐axis screw are associated with improved union rates and reduced rates of avascular necrosis when compared with traditional techniques utilizing three cannulated screws (Figure 9).^41^, ^42^

In the present study, we also compared mechanical differences between G‐ITR and G‐TRI. As reported in previous studies,^43^, ^44^ there were no significant differences in stiffness or IFM between these two groups. However, our detailed biomechanical analysis (Figure 5) revealed that the G‐TRI technique was associated with a greater ability to resist shearing forces, while the G‐ITR technique was associated with a greater ability to resist detachment forces. As expected, the two lag screws placed superiorly and inferiorly are better at resisting tensile and shearing forces, respectively. Further regression analysis revealed the advantages of G‐ITR given increases in SIM and vice versa. Interestingly, we observed that patients with higher detachment forces exhibited typical thin femoral necks (390.66 mm^2^) with the lowest neck‐shaft angle (NSA) values (121.88°), while those with higher shearing forces exhibited thick femoral necks (928.00 mm^2^) and the highest NSA values (Figure 8B). This phenomenon can be theoretically demonstrated using the derivation of the cantilever beam formula (Figure 8C), in which the length of the femoral neck exhibits a positive correlation with the maximum tensile stress, while the thickness of the femoral neck and NSA exhibit negative correlations with maximum tensile stress. Generally, for surgeons who still prefer the three‐screw technique due to concerns related to the additional screw's effect on vascular supply, our results suggest that an inverted triangular configuration should be used for patients with a thin femoral neck and lower NSA values, while a regular triangular configuration should be used for patients with a thick femoral neck and higher NSA values. However, further studies are required to verify the validity of this conclusion.

Apart from the abovementioned cannulated screw techniques, dynamic hip screws (DHS) with an anti‐rotational screw are also a common clinical strategy for vertical femoral neck fractures. This device has the advantage of providing angular stability and has been proved to be stiffer than cannulated screws in a previous biomechanical study.^45^ However, the implantation of DHS is more complicated than cannulated screws and it requires an invasive procedure with dramatic damage to the blood supply, soft tissue dissection, and large bone volume loss. Clinical studies have found a statistically higher operative time, incision size, intraoperative blood loss,^46^ and subsequently higher avascular necrosis rate^5^, ^6^ in DHS compared with cannulated screws. Consequently, cannulated screws with the advantage of being minimally invasive, easy handling, and the ability to induce dynamic compression remain the most promising fixation strategy.

The present study possesses some limitations. First, all models were developed using linear elastic materials and did not incorporate bone plastic deformation or screw loosening processes, which are known to lead to mechanical failure in older patients with osteoporotic fractures. In addition, underestimations of IFM values were attributed to the intact fracture surface and accurate anatomic reduction employed in our models. Our study also focused only on initial stability rather than that during the bone healing process. Furthermore, the thread of the implants was simplified in this study, but it has been proven to have little effect on the outcome.^47^ Despite these limitations, our findings may aid orthopedic surgeons in selecting the most appropriate fixation strategy in clinical practice.

In conclusion, our findings indicate that the techniques utilized in G‐ALP and G‐BUT provided the most stability with the least IFM in vertical femoral neck fractures. Given its unique biomechanical characteristics, relatively lower implant stress, and decreased likelihood of surgical dissection, G‐ALP may be more reliable than G‐BUT. However, augmentation using a buttress plate may be helpful in patients with comminuted fractures when adequate compression cannot be achieved using fragments alone. A regular triangular screw configuration can be used to ensure maximum anti‐shearing ability and may represent the most appropriate choice for patients with a thick femoral neck and high NSA values, while an inverted triangular configuration may be the most appropriate choice for patients with a thin femoral neck and lower NSA values.

## CONFLICT OF INTERESTS

The authors declare that there are no conflict of interests.

## ETHICS STATEMENT

CT scanning of participants was approved by the Ethics Committee of Shanghai Sixth People's Hospital (Approval No. 2016‐143) and written consent was obtained from the participant.

## AUTHOR CONTRIBUTIONS

*Design the study, computational simulation and experimental validation, analysis the results, draft the manuscript*: Dajun Jiang. *Evaluate the results and technical support, experimental validation, revise the manuscript*: Shi Zhan. *Provide clinical cases*: Lei Wang. *Revise the manuscript and language polishing*: Lewis L. Shi. *Evaluate the results*: Ming Ling. *Design the study, supervised the study, revise the manuscript*: Hai Hu. *Design the study, supervised the study, experimental validation, revise the manuscript*: Weitao Jia. All authors have read and approved the final submitted manuscript.

## Supporting information

Supporting information.
